# Prognostic models to predict survival in patients with advanced non-small cell lung cancer treated with first-line chemo- or targeted therapy

**DOI:** 10.18632/oncotarget.8309

**Published:** 2016-03-23

**Authors:** Rossana Berardi, Silvia Rinaldi, Matteo Santoni, Thomas Newsom-Davis, Michela Tiberi, Francesca Morgese, Miriam Caramanti, Agnese Savini, Consuelo Ferrini, Mariangela Torniai, Ilaria Fiordoliva, Marc Bower, Stefano Cascinu

**Affiliations:** ^1^ Clinica di Oncologia Medica, Università Politecnica delle Marche, Azienda Ospedaliero-Universitaria Ospedali Riuniti Umberto I – GM Lancisi – G Salesi di Ancona, Ancona, Italy; ^2^ Chelsea and Westminster Hospital, London, United Kingdom; ^3^ Chirurgia Toracica, Università Politecnica delle Marche, Azienda Ospedaliero-Universitaria Ospedali Riuniti Umberto I – GM Lancisi – G Salesi di Ancona, Ancona, Italy; ^4^ Oncologia Medica-Università degli Studi di Modena e Reggio Emilia, Modena, Italy

**Keywords:** lung cancer, neutrophil to lymphocyte ratio, platinum-base chemotherapy, prognosis, targeted therapy

## Abstract

**Background:**

We aimed to assess the prognostic role of neutrophilia, lymphocytopenia and the neutrophil-to-lymphocyte ratio (NLR), and to design models to define the prognosis of patients receiving first-line chemo- or targeted therapy for advanced non-small cell lung cancer (NSCLC).

**Materials and Methods:**

We retrospectively analysed 401 consecutive patients with advanced NSCLC treated with first line chemo- or targeted therapy. Patients were stratified into two groups with pre-treatment NLR ≥ 3.7 (Group A) *vs*. < 3.7 (Group B). The best NLR cut-off was identified by ROC curve analysis.

**Results:**

At baseline 264 patients had NLR≥3.7 (Group A), whilst 137 had lower NLR (Group B). Median OS was 10.8 months and 19.4 months in the two groups (*p* < 0.001), while median PFS was 3.6 months and 5.6 months, respectively (*p* = 0.012). At multivariate analysis, ECOG-PS≥2, stage IV cancer, non-adenocarcinoma histology, EGFR wild-type status and NLR were predictors of worse OS. Stage IV cancer, wild type EGFR status and NLR≥3.7 were independent prognostic factors for worse PFS. Patients were stratified according to the presence of 0-1 prognostic factors (8%), 2-3 factors (73%) and 4-5 factors (19%) and median OS in these groups was 33.7 months, 14.6 months and 6.6 months, respectively (*p* < 0.001). Similarly, patients were stratified for PFS based on the presence of 0-1 prognostic factor (15%), 2 factors (41%) and 3 factors (44%). The median PFS was 8.3 months, 4.6 months and 3.3 months respectively (*p* < 0.001).

**Conclusion:**

Pre-treatment NLR is an independent prognostic factor for patients with advanced NSCLC treated with first-line therapies.

## INTRODUCTION

Lung cancer is the leading cause of cancer related death in both men and women [1]. Non-small cell lung cancer (NSCLC) accounts for approximately 85% of all lung cancers. NSCLC is often insidious, producing no symptoms until the disease is advanced. Over the past few years a variety of prognostic and predictive factors have been investigated in patients with NSCLC, and several prognostic models proposed to predict the outcome of patients with NSCLC after surgery [2–4] or chemotherapy and/or radiotherapy [5–6]. Despite this the prognosis for patient with advanced NSCLC remains dismal, and novel approaches are required in order to optimize patient outcomes and guide treatment decisions.

Inflammation contributes to the pathogenesis and progression of lung cancer. For example, chronic inflammatory lung diseases such as sarcoidosis and chronic obstructive pulmonary disease (COPD) are associated with a higher risk of lung cancer [7], whilst the chronic use of anti-inflammatory drugs seems to reduce the risk [8]. *In vitro* studies suggest that direct cell-cell interactions between neutrophils and NSCLC cells can induce the release of inflammatory mediators, which may promote tumor cell proliferation [9]. Indeed, NSCLC cells might secret immunoreactive IL-8 and stimulate polymorphonuclear neutrophils (PMNs) to release Arginase 1. Both molecules inhibit T-cell proliferation and favour tumor cell progression [10]. An elevated neutrophil count has been associated with poor prognosis in patients with NSCLC treated with chemotherapy, with a difference in overall survival (OS) of approximately 9 months compared to those with normal neutrophil count (19.3 vs. 10.2 months) [11].

Markers of inflammation, such as the neutrophil-to-lymphocyte ratio (NLR), and their clinical significance in NSCLC patients are still under evaluation. NLR is an easily measurable parameter of systemic inflammation. Increased pre-treatment NLR has been demonstrated to be associated with poor outcome for various types of cancers including gastric cancer [12], advanced pancreatic cancer [13], hepatocellular carcinoma [14], colorectal liver metastases [15], bladder cancer [16], malignant mesothelioma [17], ovarian cancer [18] and renal cell carcinoma [19–22].

The aim of this study was to assess the prognostic role of pre-treatment neutrophilia, lymphocytopenia and NLR and to design a model to define the prognosis of patients receiving first-line chemo- or targeted therapy for advanced NSCLC.

## RESULTS

### Patient characteristics

Five hundreds and twenty-one patients were treated with first-line therapies. Of these, 401 patients (275 males and 126 female) were included in the NLR analysis, while 120 patients were excluded for the lack of data on pre-treatment NLR.

The median age was 68y (range 25−86). The majority were current or former smokers (323 patients, 81%). Histology was adenocarcinoma in 258 patients (64%), squamous carcinoma in 94 patients (23%) and other histology in 49 patients (13%). One hundred and twenty-one (30%) patients have stage III and 280 patients (70%) has stage IV disease. First-line therapy involved chemotherapy in 373 patients (93%) and EGFR-TKIs in 28 patients (7%). The complete list of patients' characteristics is summarized in Table 1.

**Table 1:** Patient characteristics

Patients	Overall401 (%)	NLR ≥ 3.7264 (66)	NLR < 3.7137 (34)	*p*
**Gender**				*0.910*
Male	275 (69)	180 (68)	95 (69)	
Female	126 (31)	84 (32)	42 (31)	
**Age, years**	68	66	68	*0.878*
Range	25−86	25−83	29−86	
**ECOG-PS ≥** 2	35 (9)	26 (10)	15 (11)	*0.732*
**ECOG-PS** < 2	366 (91)	236 (90)	122 (89)	
**Histology**				*0.312*
Adenocarcinoma	258 (64)	166 (63)	92 (67)	
Squamous carcinoma	94 (23)	61 (23)	33 (24)	
Other	49 (13)	37 (14)	12 (9)	
**Tumor Stage**				*0.819*
Stage III	121 (30)	81 (31)	40 (29)	
Stage IV	280 (70)	183 (69)	97 (71)	
**EGFR mutation status**				*0.839*
Wild-type	360 (90)	240 (91)	120 (88)	
Mutated	41 (10)	24 (9)	17 (12)	
**Smoking history**				*0.594*
Former/current smoker	323 (81)	211 (80)	113 (82)	
Never smokers	78 (19)	53 (20)	24 (18)	
**Common sites of metastasis**				*0.855*
Lung	249 (62)	182 (69)	67 (49)	
Bone	161 (40)	121 (46)	40 (29)	
Nervous system	81 (20)	61 (23)	20 (15)	
Liver	74 (18)	52 (20)	22 (16)	
**First-line therapy**				*0.687*
Platinum-based chemotherapy	311 (78)	208 (79)	103 (75)	
Non platinum-based	62 (15)	38 (14)	24 (18)	
EGFR-TKI	28 (7)	18 (7)	10 (7)	
**Response to first-line therapy**				*0.053*
Partial response	128 (32)	75 (28)	53 (38)	
Stable disease	134 (33)	88 (33)	46 (34)	
Progressive disease	139 (35)	101 (39)	38 (28)	
**Neutrophilia**	179 (45)	165 (63)	14 (10)	***< 0.001***
**Lymphocytopenia**	87 (22)	85 (32)	2 (1)	***< 0.001***

The median neutrophil count was 7020/mm³, median lymphocyte count was 1400/mm³ and median NLR was 5.1. Absolute neutrophilia (≥7500/mm^3^) was present in 179 patients (45%), while lymphocytopenia ( < 1500/mm^3^) was reported in 87 patients (22%).

**Figure 1:**
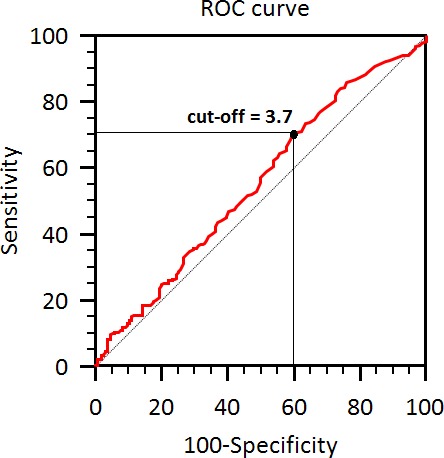
Cut-off identification by ROC curve

The best NLR cut-off was ≥ 3.7 vs. < 3.7, as identified by ROC curve analysis (Figure 1). Patients were further divided into two groups according to NLR. Two hundred and sixty-four patients (66%) had NLR ≥ 3.7 at baseline (Group A), while 137 (34%) had lower NLR (Group B).

### Overall survival (OS)

The median OS from first-line therapy was 14.4 months (95% CI 12.4 to 16.9) in the total population. Two hundred and forty eight patients died during follow-up.

The median OS was 19.6 months (95% CI 16.5 to 28.6) in the 78 non-smokers and 13.1 months (95% CI 10.8 to 16.0) in the 323 smokers (*p* = 0.08). Stratified by gender, the median OS was 11.7 months (95% CI 9.5 to 15.5) in males and 18.4 months (95% CI 14.4 to 27.9) in females (*p* = 0.005). No significant difference was found between patients aged < 70y vs. ≥70y (14.7 vs. 14.4 months, *p* = 0.335). Patients with ECOG-PS ≥ 2 had a significantly shorter OS compared to those with ECOG-PS < 2 (4.6 vs. 15.5 months, *p* < 0.001).

With respect to histology, the median OS was 11.9 months (95% CI 9.5 to 15.5) in patients with squamous carcinoma, 16.2 months (95% CI 13.4 to 22.0) for adenocarcinoma and 12.7 months (95% CI 9.6 to 16.8) for other histologies (*p* = 0.053).

**Figure 2:**
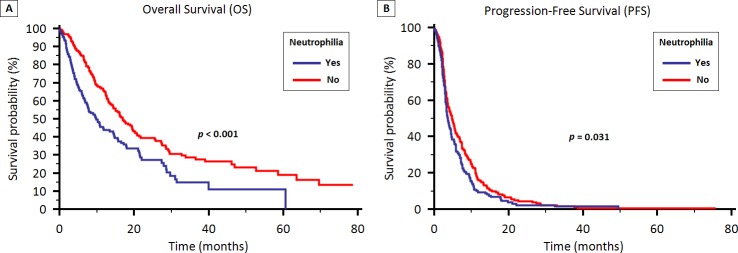
OS (2A) and PFS (2B) stratified by the presence of neutrophilia in patients treated with first-line therapy for locally advanced or metastatic NSCLC

The median OS was 9.7 months (95% CI 7.4 to 14.4) and 16.9 months (95% CI 14.4 to 20.6) for patients with and without absolute neutrophilia, respectively (*p* < 0.001) (Figure 2A). No significant difference was found between patients with and without lymphocytopenia (13.7 vs. 14.5 months, *p* = 0.319).

**Figure 3:**
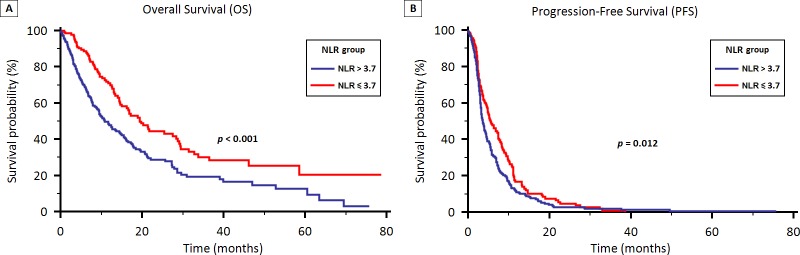
OS (3A) and PFS (3B) stratified by neutrophil to lymphocyte ratio (NLR) in patients treated with first-line therapy for locally advanced or metastatic NSCLC

Stratified by NLR ≥ 3.7 vs. < 3.7, the median OS was 10.8 months (95% CI 9.3 to 14.7) in Group A and 19.4 months (95% CI 15.0 to 29.1) in Group B (*p* < 0.001) (Figure 3A).

Univariate analysis showed that gender (male), ECOG-PS ≥ 2, current or former smokers, tumor stage IV, non-adenocarcinoma histology, wild-type EGFR status, neutrophilia and NLR ≥ 3.7 were significantly associated with worse OS (Table 2). At multivariate analysis, ECOG-PS, tumor stage, histology, EGFR status and NLR were predictors of OS (Table 2).

**Table 2:** Univariate and multivariable analysis of predictors of OS in patients treated with first-line therapy for locally advanced or metastatic NSCLC

OVERALL SURVIVAL				
	Univariate Cox Regression		Multivariable Cox regression	
	HR (95%CI)	*p-value*	HR (95%CI)	*p-value*
Age (≥ 70y vs. < 70y)	1.13 (0.88−1.45)	0.336		
Gender (F vs. M)	0.67 (0.51−0.88)	**0.005**		
ECOG-PS (≥ 2 vs. < 2)	2.50 (1.65−3.78)	**<0.001**	2.32 (1.37−3.92)	**0.002**
Smoke status (Y vs. N)	1.36 (0.96−1.91)	**0.08**		
Tumor Stage (IV vs. III)	1.60 (1.21−2.13)	**0.001**	1.56 (1.14−2.12)	**0.005**
Histology (non-AC vs. AC)	1.37 (1.07−1.77)	**0.013**	1.37 (1.02−1.82)	**0.034**
EGFR Status (WT vs. MT)	2.32 (1.15−4.67)	**0.020**	2.83 (1.14−7.01)	**0.025**
Neutrophilia (Y vs. N)	1.67 (1.30−2.15)	**<0.001**		
Lymphocytopenia (Y vs. N)	1.16 (0.86−1.57)	0.319		
NLR (≥ 3.7 vs. < 3.7)	1.74 (1.32−2.28)	**<0.001**	1.74 (1.26−2.41)	**<0.001**

AC = Adenocarcinoma; CI = confidence interval; ECOG-PS = Eastern Cooperative Oncology Group Performance Status; EGFR = Epidermal growth factor receptor; F = female; HR = hazard ratio;

### Progression-free survival (PFS) and response to first-line therapy

The median PFS was 4.1 months (95% CI 3.6 to 4.8) in the overall study population, 3.7 months (95% CI 3.3 to 4.4) in males and 5.4 months (95% CI 4.4 to 6.9) in females (*p* = 0.04).

The median PFS was 5.8 months (95% CI 3.9 to 8.7) in the 78 non-smokers and 4.1 months (95% CI 3.6 to 4.8) in the 323 smokers (*p* = 0.08). No significant difference was found with respect to age < 70y vs. ≥70y (3.9 vs. 4.2 months, *p* = 0.945), ECOG-PS ≥ 2 vs. < 2 (3.7 vs. 4.2 months, *p* = 0.10), or histology (squamous carcinoma vs. adenocarcinoma vs. other histologies: 3.9 vs. 4.4 vs. 4 months).

Median PFS was 3.6 months (95% CI 3.2 to 4.4) and 4.8 months (95% CI 3.9 to 5.6) in patients with and without absolute neutrophilia, respectively (*p* = 0.031) (Figure 2B). Similarly to OS, no difference was found between patients with and without lymphocytopenia (3.7 vs. 4.2 months, *p* = 0.42).

Stratified by NLR, the median PFS was 3.6 months (95% CI 3.2 to 4.2) in Group A and 5.6 months (95% CI 4.6 to 7.4) in Group B (*p* = 0.012) (Figure 3B).

Furthermore, univariate analysis showed that gender (male), ECOG-PS ≥ 2, current or former smokers, tumor stage IV, wild-type EGFR status, neutrophilia and NLR ≥ 3.7 were significantly associated with worst PFS (Table 3). Multivariate Cox regression analysis revealed that tumor stage IV, wild-type EGFR status and NLR ≥ 3.7 were independent prognostic factors for worse PFS (Table 3).

**Table 3:** Univariate and multivariable analysis of predictors of PFS in patients treated with first-line therapy for locally advanced or metastatic NSCLC

PROGRESSION-FREE SURVIVAL				
	Univariate Cox Regression		Multivariable Cox regression	
	HR (95%CI)	*p-value*	HR (95%CI)	*p-value*
Age (≥ 70y vs. < 70y)	1.01 (0.82−1.24)	0.945		
Gender (F vs. M)	0.80 (0.64−0.99)	**0.043**		
ECOG-PS (≥ 2 vs. < 2)	1.37 (0.94−2.00)	**0.105**		
Smoke status (Y vs. N)	1.29 (0.97−1.71)	**0.083**		
Tumor Stage (IV vs. III)	1.21 (0.97−1.52)	**0.09**	1.33 (1.04−1.70)	**0.024**
Histology (non-AC vs. AC)	0.96 (0.78−1.19)	0.727		
EGFR Status (WT vs. MT)	2.47 (1.56−3.92)	**<0.001**	2.67 (1.57−4.53)	**<0.001**
Neutrophilia (Y vs. N)	1.25 (1.02−1.54)	**0.032**		
Lymphocytopenia (Y vs. N)	1.09 (0.85−1.39)	0.518		
NLR (≥ 3.7 vs. < 3.7)	1.32 (1.06−1.64)	**0.013**	1.36 (1.04−1.76)	**0.023**

AC = Adenocarcinoma; CI = confidence interval; ECOG-PS = Eastern Cooperative Oncology Group Performance Status; EGFR = Epidermal growth factor receptor; F = female; HR = hazard ratio;

As for response to first-line therapy, we compared by chi-squared test the rate of disease progressions in patients with NLR ≥ 3.7 vs. < 3.7. In the subgroup with higher NLR, the progression rate was 81% vs 71% in patients with NLR < 3.7, showing a significant difference between these two populations (*p* = 0.03).

### Prognostic models for OS and PFS

**Figure 4:**
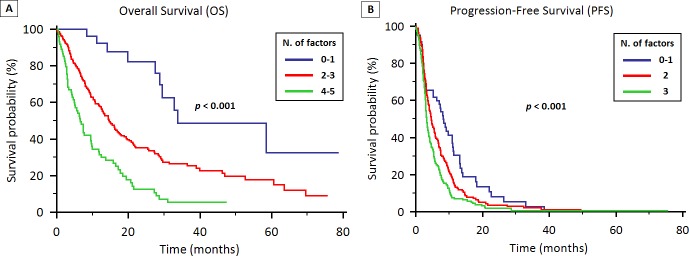
Prognostic models for OS (4A) and PFS (4B) in patients treated with first-line therapy for locally advanced or metastatic NSCLC

Based on the multivariate analysis, ECOG-PS, tumor stage, histology, EGFR status and NLR were significantly associated with OS. A prognostic model was therefore created, stratifying patients according to the presence of 0-1 of these prognostic factor (33 patients, 8%), 2-3 factors (293 patients, 73%) and 4-5 factors (75 patients, 19%). In the 3 groups, the median OS was 33.7 months (95% CI 29.5 to N.A.), 14.6 months (95% CI 12.7 to 17.4) and 6.6 months (95% CI 4.9 to 9.7), respectively (*p* < 0.001; Figure 4A).

As a sensitivity analysis, we evaluated our prognostic model also in patients with EGFR wild-type tumors. In this subgroup, OS was 32.8 (95% CI 28.3 to N.A.), 14.4 (95% CI 12.2 to 17.4) and 9.7 (95% CI 4.9 to 11.8) months in patients with 0-1, 2-3 or 4-5 factors, respectively (*p* = 0.041, Figure S1).

A similar prognostic model based on the multivariate analysis for PFS was created, with patients stratified on the presence of 0-1 factor (60 patients, 15%), 2 factors (166 patients, 41%) and 3 factors (175 patients, 44%). The median PFS was 8.3 months (95% CI 6.3 to 11.2), 4.6 months (95% CI 3.9 to 5.8) and 3.3 months (95% CI 3.0 to 3.9), respectively (*p* < 0.001; Figure 4B).

## DISCUSSION

A better understanding of the complexity of tumor-immune interactions in patients with NSCLC has facilitated an increased interest and development of immune therapeutic strategies. However the effects of chemotherapy and targeted agents on the immune system and its role in tumour response to therapies remains less clear.

Previous groups have examined pre-treatment NLR in NSCLC. In early-stage disease, NLR was positively associated with the prognosis for patients treated with stereotactic radiation [23] and surgery [24–26]. Zhang *et al*. showed that preoperative lymphocytopenia correlated with lymphatic invasion and a shorter disease-free survival (DFS, 318 vs. 669 days) in 142 patients with NSCLC who underwent lobectomy and lymph node dissection and adjuvant chemotherapy. Neutrophil count however was not associated with DFS [27]. Elevated pre-treatment NLR was significantly associated with worse OS in 81 advanced EGFR-mutated NSCLC patients treated with first-line EGFR TKIs [28], as well as in 199 never smokers with advanced NSCLC receiving gefitinib or standard chemotherapy [29]. In addition, Yao *et al.* found that high NLR was associated with shorter OS and PFS in patients with advanced NSCLC treated with first-line platinum-based chemotherapy [30]. More recently, three different meta-analyses have confirmed the prognostic role of NLR in patients with lung cancer [31–33].

To the best of our knowledge, this is the largest study investigating for the role of neutrophilia, lymphocytopenia and NLR in patients with NSCLC treated with first-line therapies. In our study, ECOG-PS ≥ 2, IV tumor stage, non-adenocarcinoma histology, EGFR wild-type status and NLR were predictors of worse OS, whilst tumor stage IV, wild-type EGFR status and NLR ≥ 3.7 were independent prognostic factors for worse PFS. Based on these data, we designed two prognostic models for OS and PFS. Median OS differed according to the presence of 0-1, 2-3 or 4-5 prognostic factors, thus identifying patients with good, intermediate and poor prognosis (33.7 months vs. 14.6 months vs. 6.6 months, *p* < 0.001). Similarly, when patients were stratified according to the presence of 0-1, 2 or 3 prognostic factors for PFS, different duration of response to first-line therapies became evident (8.3 months vs. 4.6 months vs. 3.3 months, *p* < 0.001).

There are some limitations to this study. First, this is a retrospective study, which is susceptible to bias in data selection and analysis. Other inflammatory markers such as C-reactive protein (CRP) or procalcitonin, are not routinely measured in our institutions and so were not included. Furthermore, NLR differs among individuals and can present fluctuations due to concurrent infections and other medications, factors that cannot be fully accounted for in this study. Moreover, some patients (13/41) with EGFR mutated tumors were not treated with EGFR-TKIs as first-line therapy due to unavailable data on EGFR mutational status at time of the beginning of treatment.

Despite these limitations, our study suggests that pre-treatment NLR is associated with PFS and OS in patients treated with first-line therapies for advanced NSCLC. Prospective studies are needed to evaluate and validate the prognostic models described here and adequately assess the potential role of NLR in guiding treatment decisions, patient selection, and clinical trials design.

## MATERIALS AND METHODS

### Study population and data collection

The study population was adults with a histological or cytological diagnosis of locally advanced or metastatic NSCLC treated with first-line chemotherapy or targeted therapy according to EGFR mutational status at two institutions (Università Politecnica Marche, Italy and Chelsea & Westminster Hospital, UK) between 1^st^ May 2009 and 31th October 2014. Tumor stages were assessed according to the tumor-node-metastasis (TNM) criteria and included patients with stage IIIB and IV, as well as patients in stage IIIA not suitable for surgery, as defined in AJCC version 7 [34].

Patients were ineligible if they had received surgery or radiotherapy within one month from the start of first-line therapy and if they presented factors that could influence NLR (see below). Data was retrospectively collected from patients' medical records.

Treatment with first-line chemotherapy or TKIs was continued until evidence of disease progression on scans, unacceptable adverse events, or death. Follow-up generally consisted of regular physical examination and laboratory assessment (hematologic and serum biochemical measurements), and imaging studies by computed tomography (CT) or magnetic resonance imaging (MRI) scans was carried out according to local procedures every 8-12 weeks.

The OS was defined as the time from the beginning of first-line treatment until death from any cause. Progression free survival (PFS) was defined as the time from beginning of treatment to disease progression or death from any cause. Patients without tumour progression or death at the time of the data cut-off for the analysis or at the time of receiving an additional anticancer therapy were censored at their last date of adequate tumour evaluation.

Peripheral blood samples were obtained 1 to 7 days before the start of first-line therapy. Patients without available data on pre-treatment NLR and those with baseline comorbidities that might influence NLR, such as chronic lymphocytic leukaemia (CLL), chronic inflammatory diseases, and recent signs of infection or therapy with steroids or granulocyte colony stimulating factor (G-CSF), were excluded.

### Statistical analysis

PFS and OS were estimated using Kaplan-Meier method with Rothman's 95% confidence intervals (CI) and compared across the groups using the log-rank test. Patients with a stable disease (SD), partial remission, and a complete remission were considered as responders.

Pre-treatment NLR was calculated by dividing the absolute neutrophil count by the absolute lymphocyte count and potential factors associated with outcome were evaluated, including patients' age (≥ 70y vs. < 70y), gender, tumor stage, histology, EGFR mutational status, Eastern Cooperative Oncology Group-Performance Status (ECOG-PS), smoking history, neutrophil count, lymphocyte count, and NLR. We determined by ROC analysis the value that best discriminated between good and poor survival.

Cox proportional hazards models were applied to explore patients' characteristics predictors of survival in univariate- and multivariable analysis. Variables not fitting at univariate analysis were excluded from the multivariate model. No-multicollinearity of the grouped co-variates was checked. Significance level in the univariate model for inclusion in the multivariate final model was more liberally set at a 0.2 level, according to Hosmer *et al*. [35, 36]. The likelihood ratio test was conducted to evaluate the improvement in prediction performance gained by backward elimination of variables from the prognostic model [37]. All other significance levels were set at a 0.05 value and all *P* values were two-sided. Statistical analyses were performed using MedCalc version 11.4.4.0 (MedCalc Software, Broekstraat 52, 9030 Mariakerke, Belgium).

## SUPPLEMENTARY MATERIAL FIGURES


