# Supplementary material for: Prognostic models to predict survival in patients with advanced non-small cell lung cancer treated with first-line chemo- or targeted therapy
Source: Oncotarget. 2016 Mar 23;7(18):26916–24. doi: 10.18632/oncotarget.8309 (PMC5042025; doi:10.18632/oncotarget.8309)
Supplement: Supplementary file 1 [file oncotarget-07-26916-s001.pdf]

Prognostic models to predict survival in patients with advanced non-small cell lung cancer treated with first-line chemo- or targeted therapy

Supplementary Material

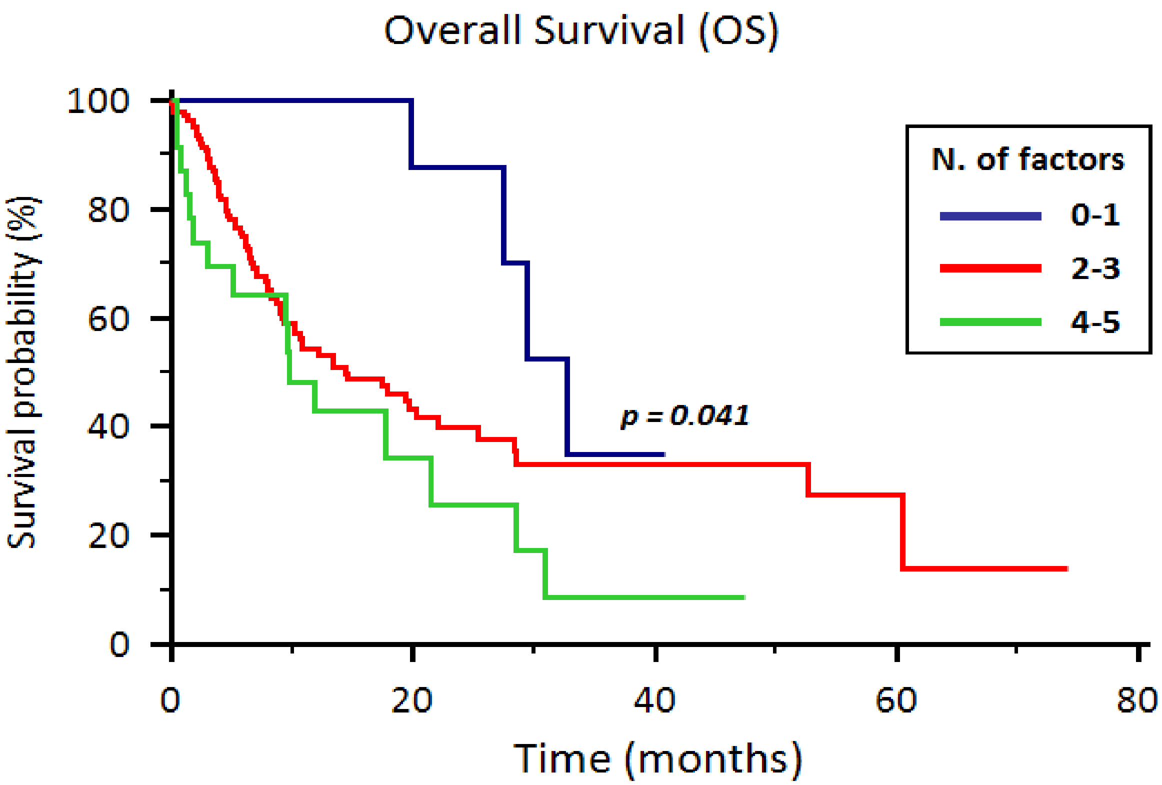

Figure S1. Prognostic model for OS in patients with EGFR wild-type locally advanced or metastatic NSCLC treated with first-line therapy.
